# Mitochondria: A Common Target for Genetic Mutations and Environmental Toxicants in Parkinson’s Disease

**DOI:** 10.3389/fgene.2017.00177

**Published:** 2017-11-17

**Authors:** Martin P. Helley, Jennifer Pinnell, Carolina Sportelli, Kim Tieu

**Affiliations:** ^1^Department of Environmental Health Sciences, Florida International University, Miami, FL, United States; ^2^Peninsula Schools of Medicine and Dentistry, Plymouth University, Plymouth, United Kingdom

**Keywords:** Parkinson’s disease, mitochondrial dynamics, mitochondrial dysfunction, neurodegeneration, neurotoxicity, environmental toxins, gene–environment interaction

## Abstract

Parkinson’s disease (PD) is a devastating neurological movement disorder. Since its first discovery 200 years ago, genetic and environmental factors have been identified to play a role in PD development and progression. Although genetic studies have been the predominant driving force in PD research over the last few decades, currently only a small fraction of PD cases can be directly linked to monogenic mutations. The remaining cases have been attributed to other risk associated genes, environmental exposures and gene–environment interactions, making PD a multifactorial disorder with a complex etiology. However, enormous efforts from global research have yielded significant insights into pathogenic mechanisms and potential therapeutic targets for PD. This review will highlight mitochondrial dysfunction as a common pathway involved in both genetic mutations and environmental toxicants linked to PD.

## Introduction

Parkinson’s disease (PD), characterized by degeneration of the nigrostriatal dopaminergic pathway, is the second most prevalent neurodegenerative disorder after Alzheimer’s disease. Pathological hallmarks of PD include the loss of dopaminergic neurons in the substantia nigra pars compacta (SNpc) and the presence of cytoplasmic protein aggregates known as Lewy bodies in remaining dopaminergic neurons (Przedborski, 2017). When degeneration in these neurons results in a threshold reduction of approximately 80% of striatal dopamine, motor symptoms of PD such as rigidity, resting tremor, bradykinesia and postural instability start to emerge (Dauer and Przedborski, 2003). PD is a complex and multifactorial brain disorder. Approximately 30% of the familial and 3–5% of sporadic PD cases are caused by monogenic mutations, while the other remaining cases are classified as idiopathic and sporadic with unknown etiology (Klein and Westenberger, 2012; Hernandez et al., 2016). Environmental factors and gene–environment interactions have been implicated in idiopathic PD. This neurological disorder is therefore a polygenic disease with various genetic and environmental contributors cumulatively directing its pathological development.

Since the identification of the first missense substitution in *SNCA* (Polymeropoulos et al., 1997), the gene encoding α-synuclein, the understanding of the genetic contribution to PD has progressed rapidly and has become rather complex. In addition to the emergence of multiple loci as causative factors in familial PD (**Table 1**), genome wide association studies (GWAS) and Genome-wide complex trait analysis (GCTA) have uncovered a significant genetic component to the idiopathic disease (Hernandez et al., 2016; Przedborski, 2017). Although mutations in these genes alone may not be considered as causative, they significantly increase the risk of developing idiopathic PD. This is, at least in part, due to incomplete and variable penetrance of the majority of these mutations. The emergence of genetic risk factors for cases of idiopathic PD raises an important question: What is causing the increased susceptibility to the disease in individuals that carry these mutations? One possible and particularly pertinent explanation is that risk factor mutations render the individual more sensitive to the pathological influence of environmental factors thus bringing the idea of gene–environment interactions to the forefront. This review will discuss the impact of genetic mutations, environmental toxicants and gene–environment interactions on PD pathogenesis. Furthermore, we will highlight mitochondria as a common target for both genes and environmental toxicants linked to PD.

**Table 1:** Major monogenic mutations and associated risk genes in PD.

Gene	PARK Locus	Gene Locus	Inheritance	Mutations	Prevalence	First reference linked to PD	Method of identification
*SNCA*	*PARK 1/4*	*4q21-22*	AD	A53T, A30P, E46K, G51D, H50Q, duplications, triplications	Rare, A53T is most frequent but only found in seven families worldwide	Polymeropoulos et al., 1997; Krüger et al., 1998; Singleton et al., 2003; Zarranz et al., 2004; Appel-Cresswell et al., 2013; Lesage et al., 2013	GW-Linkage
*LRRK2*	*PARK 8*	*12q12*	AD	>100 missense and non-sense mutations high risk variants, >15 of which are pathogenic	Upto 40% of familial and up to 10% of sporadic cases	Funayama et al., 2002	GW-Linkage
*VPS35*	*PARK 17*	*16q11.2*	AD	D620N	1% of familial and 0.2% of sporadic cases	Vilarino-Guell et al., 2011; Zimprich et al., 2011	Exome sequencing
*CHCHD2*	*PARK 22*	*7q11.2*	AD	10 mutations (see Koschmidder et al., 2016)	Not available	Funayama et al., 2015; Ogaki et al., 2015	GW-Linkage
*GBA*	*N/A*	*1q21*	AD	>300 mutations, L444P and N370S are most common	Varies in different PD populations but up to 31% in Ashkenazi Jewish PD patients	Tsuji et al., 1987; Neudorfer et al., 1996	Candidate gene
*Parkin*	*PARK 2*	*1p35-36*	AR	∼147 exonic mutations	77% familial EOPD and 10–20% EOPD in general	Kitada et al., 1998	GW-Linkage
*PINK1*	*PARK 6*	*6q25.2-27*	AR	>60 mutations,	>9% EOPD	Valente et al., 2004a	GW-Linkage
*DJ-1*	*PARK 7*	*1p36*	AR	>10 mutations	1–2% EOPD	Bonifati et al., 2003	GW-Linkage
*ATP13A2*	*PARK 9*	*1p36*	AR	>10 mutations	Rare (found in 11 families)	Ramirez et al., 2006; Di Fonzo et al., 2007	GW-Linkage
*FBXO7*	*PARK 15*	*22q12-13*	AR	R378G, R498X, T22M, L34R	Rare	Shojaee et al., 2008	GW-Linkage
*PLA2G6*	*PARK 14*	*22q13.1*	AR	R741Q, R747W and more	Rare	Paisan-Ruiz et al., 2009	GW-Linkage

*EOPD, Early-onset PD (Age at onset <50 years old); LOPD, Late-onset PD (Age at onset ≥ 50 years old); AD, Autosomal dominant; AR, Autosomal recessive; GW, Genome-Wide.*

## Genetic Mutations In PD

### Autosomal-Dominant Genes

#### SNCA

Perhaps the most researched PD-linked gene to date is *SNCA*, which encodes α-synuclein. While mutations in this gene are rare, the *SNCA* locus is appreciated to be a significant variant factor for PD development (Lill et al., 2012). The missense mutation α-synuclein-A53T (Polymeropoulos et al., 1997) was the first gene to be attributed to disease development and its discovery represents a defining moment in PD research, not only preceding the identification of many other genetic determinants of PD but also mechanisms of sporadic disease. Since then, other point mutations in α-synuclein (A30P, E46K, G51D, and H50Q), as well as gene duplications and triplications, have been identified in familial PD (Krüger et al., 1998; Singleton et al., 2003; Zarranz et al., 2004; Appel-Cresswell et al., 2013; Lesage et al., 2013). The fact that duplications and triplications of *SNCA* can also cause PD (Singleton et al., 2003) is significant because it indicates that elevated wild type α-synuclein alone is sufficient to cause disease. So far, A53T has been found in seven families worldwide and only one family for each other four missense mutations. Duplications are more common than triplications, which has been found in several families (Hernandez et al., 2016). α-synuclein is also understood to be a key factor of sporadic PD and present in Lewy bodies, which are abnormal proteins commonly observed in PD (Spillantini et al., 1998). α-synuclein-associated mechanisms have therefore been at the forefront of the PD research and multiple high profile discoveries have greatly contributed to the understanding of disease pathology. For example, as discussed below, the discovery that α-synuclein pathology can spread from one cell to another in a prion-like fashion has provided a key insight into how PD may develop and provide novel therapeutic strategies.

α-synuclein is a pre-synaptic protein that plays a role in SNARE complex assembly and the exocytosis of neurotransmitters (Burré et al., 2010; Garcia-Reitböck et al., 2010; Bendor et al., 2013). The pathology of α-synuclein is largely due to its propensity to aggregate; gradually transitioning from small soluble oligomers to larger insoluble fibrils, ultimately forming Lewy bodies. Although the majority of α-synuclein is present in the cytosol, the protein also localizes to mitochondria and induces dysfunction (Devi et al., 2008; Parihar et al., 2008; Liu G. et al., 2009; Nakamura et al., 2011; Subramaniam et al., 2014; Chen et al., 2015; Di Maio et al., 2016). Multiple mitochondrial targeting domains have been identified within the N-terminal domain of the protein and confer its ability to bind to components of the mitochondrial membrane (Devi et al., 2008). Specifically, α-synuclein can bind to cardiolipin (Nakamura et al., 2008), TOM20 (Di Maio et al., 2016), TOM40 (Devi et al., 2008) and VDAC (Rostovtseva et al., 2015) either to directly promote dysfunction at the membrane level or to allow import into other mitochondrial compartments. The importance of the N-terminal domain in mitochondrial targeting is emphasized by the fact that α-synuclein-A53T, a mutant with a substitution in the N-terminal region, has a greater binding affinity for mitochondria than the wild type protein (Devi et al., 2008). However, more recently it has also been argued that, rather than localizing to the mitochondria, α-synuclein specifically targets the mitochondria-associated endoplasmic reticulum membrane (MAM) where it plays a role in regulating mitochondrial morphology (Guardia-Laguarta et al., 2014).

Recent studies have demonstrated that α-synuclein induces mitochondrial dysfunction *in vivo*. Mice with global overexpression of human wild type α-synuclein in the brain using the Thy1 promoter exhibited age dependent accumulation of α-synuclein in mitochondria of the nigrostriatal dopaminergic pathway, impaired electron transport chain function and enhanced oxidative stress (Subramaniam et al., 2014). Mice with inducible α-synuclein-A53T exhibited severe mitochondrial fragmentation that preceded dopaminergic neurodegeneration and other pathologies (Chen et al., 2015). These detrimental effects may be caused by the direct and specific binding between α-synuclein and outer mitochondrial membrane components such as TOM20 and VDAC (Rostovtseva et al., 2015; Di Maio et al., 2016). Di Maio et al. (2016) demonstrated that oligomeric, dopamine-modified and phosphorylated species of α-synuclein bound to TOM20 and prevented the import of multiple mitochondrial proteins leading to mitochondrial dysfunction. Similarly, binding of wild-type α-synuclein to VDAC was sufficient to block the channel preventing bi-directional metabolite transfer thus promoting dysfunction (Rostovtseva et al., 2015). In addition to blocking the channel, α-synuclein was able to translocate through VDAC to gain access to inner mitochondrial compartments, many of which being direct pathological targets, including respiratory complex components. A similar relationship has been reported for TOM40 as antibodies against this channel prevented the mitochondrial import of α-synuclein (Devi et al., 2008). In addition to these detrimental effects on mitochondrial function, α-synuclein also interferes with normal mitochondrial dynamics (fission, fusion, and transport). Overexpression of both wild type and mutated forms of α-synuclein is associated with severe fragmentation and reduced motility *in vitro* and *in vivo* through direct and indirect mechanisms (Kamp et al., 2010; Nakamura et al., 2011; Gui et al., 2012; Guardia-Laguarta et al., 2014; Chen et al., 2015; Bido et al., 2017). Another negative impact of α-synuclein on mitochondria is its ability to reduce mitochondrial biogenesis (Zheng et al., 2010; Siddiqui et al., 2012). Following the onset of oxidative stress, α-synuclein can bind to the promoter region of Peroxisome proliferator-activated receptor gamma coactivator 1-alpha (PGC1α), a key component of mitochondrial biogenesis, and inhibit the expression of downstream genes (Siddiqui et al., 2012). Lastly, multiple α-synuclein species may also promote the formation of dysfunctional mitochondria by increasing the prevalence of mitochondrial DNA mutations (Martin et al., 2006; Bendor et al., 2013).

In addition to these direct effects, α-synuclein can also affect mitochondrial function through indirect mechanisms. For example, it is increasingly evident that mitochondrial function and autophagy are tightly linked. A reduced autophagic capacity has been identified in many neurodegenerative diseases, including PD. Many of the genes implicated in familial PD, including *SNCA, PINK1, Parkin, LRRK2, DJ-1* and glucocerebrosidase *(GBA)*, have functions related to autophagy and mitophagy, emphasizing the importance of this pathway in the disease. Mitochondrial dysfunction may be able to inhibit the autophagy pathway, perhaps by a reduction in ATP availability and increased reactive oxygen species (ROS). Equally, impaired autophagy can promote mitochondrial dysfunction by the reduced clearance of damaged, dysfunctional organelles. The complex relationship between these two cellular pathways is not fully understood, particularly regarding the question of whether mitochondrial dysfunction precedes impaired autophagy or vice versa. Regardless, the interaction between these two fundamental processes represents a critical area of future research in the neurodegenerative field.

As mentioned above, α-synuclein has a direct interaction with the autophagy pathway. Overexpression of wild type α-synuclein was shown to directly modulate autophagy via inhibition of Rab1a protein, which is important for autophagosome formation (Winslow et al., 2010). Impaired autophagy has been demonstrated *in vivo* using mice overexpressing wild-type α-synuclein (Ebrahimi-Fakhari et al., 2011) or A53T α-synuclein (Yu et al., 2009; Chen et al., 2015). Conversely, an impairment in autophagy pathways promotes the formation and accumulation of higher order α-synuclein species, thus creating a cycle of neurotoxicity (Xilouri et al., 2016). In summary, α-synuclein can impair mitochondrial function at multiple levels: inhibition of the electron transport chain, imbalanced mitochondrial dynamics, reduced mitochondrial biogenesis and autophagy blockade.

Emerging evidence indicates that α-synuclein can spread inter-cellularly through multiple mechanisms, including non-classical exocytosis, exosomal release and nanotubes that directly connect the two cells (Guo and Lee, 2014). According to the Braak staging hypothesis (Hawkes et al., 2007), the olfactory bulb and the gut are the initial spreading sites of misfolded α-synuclein. Consistent with this hypothesis, accumulation of α-synuclein has been identified in enteric neurons of patients with early-stage PD (Hilton et al., 2014). Mitochondria have been implicated in the pathological spread in neurons of both the enteric and central nervous systems. For example, structural mitochondrial abnormalities have been identified in enteric neurons of post-mortem tissue from PD patients (Baumuratov et al., 2016). In primary human fetal enteric neurons, exposure to recombinant α-synuclein induces mitochondrial dysfunction involving a reduction in complex I activity and impairment of mitochondrial respiration (Braidy et al., 2014). Exposure of enteric neurons to the environmental neurotoxin, rotenone, promotes the release of α-synuclein species which are subsequently taken up by and form aggregates in second-order neurons (Pan-Montojo et al., 2012). Exposure of rat ventral midbrain neurons to exogenous, pre-formed fibrillar structures of α-synuclein, often used to study the transmission of the protein, also produce mitochondrial dysfunction (Tapias et al., 2017). These studies, and others, suggest that mitochondria may play a central role in the spread of α-synuclein from one neuron to another. That said, however, the topic of whether α-synuclein is transmitted trans-synaptically in PD is still a topic of debate (Brundin and Melki, 2017; Surmeier et al., 2017), which is beyond the scope of this article.

#### LRRK2

First identified in a family of Japanese origin (Funayama et al., 2002) and subsequently independently confirmed in several other families from different countries, leucine-rich repeat kinase 2 (LRRK2) mutations are the most common group of mutations in PD cases (Cookson, 2015). Although highly dependent on ethnicity, LRRK2 mutations are estimated to account for up to 40% of familial cases, depending on the ethnic background, and up to 10% of sporadic PD cases worldwide (Hernandez et al., 2016), producing a disease phenotype that is similar to that of classical late-onset idiopathic PD. The most common and well characterized LRRK2 mutation, G2019S, alone is suggested to account for 4% of familial and 1% of sporadic cases. All described LRRK2 mutations, to date, affect a central enzymatic region of the protein consisting of a GTPase and kinase domain and confer a gain of function (West et al., 2007). Although more than 100 LRRK2 mutations have been reported, only a few such as (N1437H, R1441C, R1441G, R1441H, Y1699C, G2019S, and I2020T0) have been proven to cause PD and two have been nominated as risk factors (R1628P and G2385R) (Dächsel and Farrer, 2010; Paisan-Ruiz et al., 2013). The incomplete penetrance of multiple mutations has led to LRRK2 being identified as a risk factor for sporadic PD (Nalls et al., 2014; Hernandez et al., 2016). Sporadic PD patient derived cells also display a slightly increased basal activity of LRRK2, however, it is not known whether this is causative or a consequence of pre-existing pathology (Esteves et al., 2015). Interestingly, a recent study demonstrated that variability within the *DNM3* gene influences the age of onset in G2019S carriers (Trinh et al., 2016). The presence of other genetic modifiers may therefore influence disease manifestation in the case of LRRK2 mutations.

The complex physiological roles of LRRK2 are not fully understood; however, it is associated with many cellular functions, particularly those involving membrane dynamics, including autophagy, cytoskeletal dynamics, vesicle dynamics and mitochondrial function. Attempts to better understand how LRRK2 mutations contribute toward PD pathology have been restricted partly due to the difficulty in creating a LRRK2 transgenic model that recapitulates important features of PD. The majority of mutant LRRK2 transgenic models do not display appreciable dopaminergic neurodegeneration, but do exhibit multiple synaptic deficits including reduced vesicle endocytosis (Arranz et al., 2015) and altered dopamine release, uptake and signaling (Li et al., 2009; Tong et al., 2009; Melrose et al., 2010; Walker et al., 2014; Beccano-Kelly et al., 2015; Yue et al., 2015). LRRK2 has been shown to impair mitochondrial dynamics by physically interacting with Drp1 and promoting mitochondrial fragmentation associated with enhanced mitochondrial fission, increased ROS generation and impaired autophagy (Niu et al., 2012; Wang et al., 2012; Stafa et al., 2014; Esteves et al., 2015). In addition to increased Drp1 expression, a concomitant increase in Drp1-S616 phosphorylation has been reported. This specific modification is a marker for increased Drp1 activity (Wang et al., 2012; Stafa et al., 2014; Esteves et al., 2015). Overexpression of the gain of function mutations G2019S and R1441C resulted in a reduction of mitochondrial membrane potential, reduced complex IV activity, increased uncoupling, altered mitochondrial motility, and an imbalance in calcium signaling (Cooper et al., 2012; Papkovskaia et al., 2012; Cherra et al., 2013; Godena et al., 2014). Ultrastructural studies show an accumulation of condensed, disorganized and damaged mitochondria consistent with deficits in mitophagy and mitochondrial dynamics (Ramonet et al., 2011; Yue et al., 2015). Finally, increased mtDNA mutations have been identified in cerebrospinal fluid (CSF) and iPSCs derived from patients with the G2019S mutation (Sanders et al., 2014; Podlesniy et al., 2016). Taken together, although the mechanism is not entirely clear, a toxic gain of kinase function in LRRK2 mutations negatively affects mitochondrial function.

Mitochondrial dysfunction induced by LRRK2 also provides a strong link to the impaired autophagy pathway in multiple models. For example, an accumulation in autophagic and lysosomal structures occur both *in vitro* and *in vivo* caused by overexpression of PD-associated mutations (MacLeod et al., 2006; Plowey et al., 2008; Gomez-Suaga et al., 2012). A block in autophagy flux, increase in p62 levels and an accumulation of autophagosomes have been demonstrated in dopaminergic neurons generated from PD patient-derived induced pluripotent stem cells (iPSCs) expressing LRRK2-G2019S at endogenous levels (Sánchez-Danés et al., 2012). Importantly, the inhibition of mitochondrial fission, using the Drp1 peptide inhibitor P110, was shown to improve autophagy and reduce detrimental changes in cell morphology caused by the G2019S mutation in PD patient fibroblasts and dopaminergic neurons derived from iPSCs (Su and Qi, 2013). Conversely, another study showed that disrupted mitochondrial motility and an increased vulnerability to multiple stressors induced by G2019S and R1441C mutations in PD patient iPSCs was rescued by the autophagy promoter rapamycin (Cooper et al., 2012) Together, these studies suggest that autophagy and mitochondrial function are bi-directionally regulating one another.

#### GBA

An interest in GBA as a causative factor of PD followed the clinical observations that some Gaucher’s disease patients developed Parkinsonian symptoms (Neudorfer et al., 1996) and that relatives of these patients had an increased risk of developing PD (Halperin et al., 2006). Gaucher’s disease (GD, Tsuji et al., 1987) is the most common lysosomal storage disorder and is caused by homozygous and compound heterozygous mutations in the *GBA* gene. GBA is a lysosomal hydrolase, specifically a GBA which acts in the lysosomal membrane to convert the sphingolipid glucosylceramide to glucose and ceramide. Approximately 300 different mutations in *GBA* have been identified with many shown to have reduced GBA activity. Such reduction has indeed been identified in cases of PD with *GBA* mutations but, importantly, it has also been identified in the substantia nigra of sporadic PD brains (Gegg et al., 2012). More severe mutations, such as L444P, increase the risk of developing PD and promote an earlier onset as well as greater cognitive decline when compared to milder mutations, such as N370S. However, the overall mortality rate between these two mutations is not significantly different (Gan-Or et al., 2015; Arkadir et al., 2016; Cilia et al., 2016); therefore suggesting that even a partial loss of function is sufficient to cause the disease. The prevalence of *GBA* mutations varies in different PD populations but is accountable for up to 31% of PD patients with Ashkenazi Jewish ancestry (Sidransky and Lopez, 2012).

A reduction in GBA activity has been associated with mitochondrial dysfunction. GBA inhibition, either pharmacologically or genetically result in a reduction of membrane potential and ATP production, as well as an increase in oxidative stress and mitochondrial fragmentation *in vitro* (Cleeter et al., 2013). These findings have been confirmed using both fibroblasts isolated from GD patients and mouse models of GD. Fibroblasts from GD patients, with an L444P mutation, exhibited a reduction in complex I-III activity, CoQ10 expression, membrane potential and ATP production as well as an increase in oxidative stress (de la Mata et al., 2015). Mouse models demonstrate a reduction in membrane potential, complex I-III activity, oxygen consumption and an increase in mitochondrial fragmentation in neurons and glia (Osellame et al., 2013; Xu et al., 2014). Further, ultrastructural analysis of mitochondrial morphology showed impaired cristae organization, with mitochondria appearing more rounded and electron dense (Xu et al., 2014).

While the mechanisms of mitochondrial dysfunction associated with GBA mutations are unclear, an impairment of mitophagy is suggested to play an important role. This is perhaps not surprising given the role of GBA in the cell and this observation further strengthens the link between autophagy and mitochondria. In mouse models and primary fibroblasts, decreased mitophagy flux accompanied the observed mitochondrial dysfunction (de la Mata et al., 2015). However, whether an impaired mitophagy response causes the observed mitochondrial dysfunction in these models or whether it is a consequence of an upstream effect is not understood. Another contributing factor to mitochondrial dysfunction in these models is neuroinflammation. In *GBA* knockout mouse models, activation of both astrocytes and microglia precedes disease manifestation (Enquist et al., 2007; Farfel-Becker et al., 2011). Astrogliosis has also been identified in some GD patients that exhibit parkinsonian symptoms (Wong et al., 2004). It is important to note that the majority of the studies related to GBA discussed in this review have been performed in the setting of GD rather than PD. Therefore, the conclusions made from this work, although relevant, may not be directly transferrable to cases of PD with *GBA* mutations.

GBA activity appears to be linked to the expression and aggregation of α-synuclein. An accumulation of aggregated α-synuclein, β-amyloid and amyloid precursor protein (APP) in the cortex, hippocampus, striatum and substantia nigra of *GBA* mutant mice was observed (Xu et al., 2014). Further, inhibiting GBA, using zinc finger techniques, promotes the intercellular transmission of α-synuclein species and therefore contributes toward the spread of PD pathology (Bae et al., 2014). These observations are consistent with the role of GBA in autophagy function. An increase in α-synuclein expression has also been shown to inhibit GBA activity (Mazzulli et al., 2011; Gegg et al., 2012; Murphy et al., 2014), thus further impairing the clearance of α-synuclein mediated by autophagy.

#### VPS35

A single mutation, D620N, in the gene encoding vacuolar protein sorting 35 (VPS35) causes the development of an autosomal dominant, late-onset PD (Vilarino-Guell et al., 2011; Zimprich et al., 2011; Sharma et al., 2012). A number of other mutations in the gene have been identified; however, their pathological relevance is yet to be demonstrated. *VPS35* mutations are rare and only account for approximately 1% of familial and 0.2% of sporadic PD cases.

VPS35 is a core subunit of a heteropentameric complex known as the retromer, which plays an important role in the transport of endosomes to the golgi apparatus and the plasma membrane, as well as in recycling of transmembrane protein cargo (Williams et al., 2017). In recent years VPS35 has also been reported to mediate the shuttling of cargo from mitochondria to peroxisomes and lysosomes through mitochondria-derived vesicles (Braschi et al., 2010; Sugiura et al., 2014). Models of *VPS35* mutations successfully demonstrate dopaminergic neurodegeneration (Tsika et al., 2014; Wang et al., 2014, 2016; Tang et al., 2015). The pathogenic mechanism by which VPS35 induces PD is not clear, however, recent studies show that its mutation caused mitochondrial fragmentation, impaired mitochondrial function, increased ROS, α-synuclein accumulation and cell death (Tang et al., 2015; Wang et al., 2016). The D260N mutant has an increased binding interaction with Drp1, an effect that is further promoted by oxidative stress, resulting in an increased turnover of mitochondrial Drp1 complexes via their mitochondria derived vesicular trafficking to the lysosome for degradation (Wang et al., 2016). In addition to increasing mitochondrial fission, VPS35 deficiency or mutation induces mitochondrial fragmentation by impairing mitochondrial fusion (Tang et al., 2015) via mitochondrial E3 ubiquitin ligase 1 (MUL1) upregulation, leading to increased ubiquitination and proteasomal degradation of the fusion protein MFN2. Together, these studies strongly support the role of VPS35 in imbalanced mitochondrial dynamics.

#### CHCHD2

Although little is known about the physiological roles of Coiled-Coil-Helix-Coiled-Coil-Helix Domain Containing 2 (CHCHD2) or its role in PD pathology, several mutations in the *CHCHD2* gene cause an autosomal dominant form of late-onset PD and dementia with Lewy bodies (Funayama et al., 2015; Ogaki et al., 2015; Koschmidder et al., 2016). CHCHD2 contains a mitochondrial targeting sequence in the N-terminal domain and localizes to intermembrane space of mitochondria, where it binds to the complex IV (cytochrome c oxidase, COX). This binding is required for COX activity (Aras et al., 2015). CHCHD2 is therefore necessary for oxidative phosphorylation (Baughman et al., 2009). Indeed, reduced CHCHD2 expression results in reduced COX activity, collapsed membrane potential and an increase in ROS and mitochondrial fragmentation (Aras et al., 2015), resulting in cell death in *Drosophila* (Meng et al., 2017). PD-related mutant flies exhibited a chronic state of oxidative stress, reduced oxygen consumption, ATP production, increased cytochrome c release and displayed mitochondria with abnormal ultrastructure leading to degeneration of dopaminergic neurons. This study also suggests that CHCHD2 may be an important mitochondrial stress response protein and can modulate cell death signaling by interacting with cytochrome c and MICS1, a member of the Bax inhibitor-1 superfamily (Meng et al., 2017). Although CHCHD2 predominantly localizes in mitochondria and directly binds to complex IV, CHCHD2 is also a transcription factor of the complex IV subunit 4 isoform COX4I2 (Aras et al., 2015). Because of this unique feature, loss of CHCHD2 function negatively affects complex IV both functionally and structurally.

### Autosomal-Recessive Genes

#### *PINK1* and *Parkin*

Mutations in PTEN-induced kinase 1 (*PINK1*) and *Parkin* both cause an almost identical form of autosomal recessive, early onset familial PD which can present with or without Lewy Bodies (Kitada et al., 1998; Sasaki et al., 2004; Valente et al., 2004a; Samaranch et al., 2010). A mutation in *Parkin* was first identified in a Japanese family (Matsumine et al., 1997; Kitada et al., 1998) and are now collectively shown to encompass a wide range of variations spanning the entire length of the gene conferring a loss of function (Klein and Westenberger, 2012). *Parkin* mutations are considered as the most prevalent autosomal recessive mutations in PD accounting for approximately up to 77% of familial early-onset PD and 10–20% of early-onset PD in general (Klein and Lohmann-Hedrich, 2007; Klein and Westenberger, 2012; Kilarski et al., 2012). Mutations in *PINK1*, first identified in an Italian family (Valente et al., 2001), are the second most common cause of early-onset autosomal recessive PD accounting for up to 9% of cases (Klein and Westenberger, 2012). Mutations span the length of *PINK1*, with the majority being missense or non-sense mutations and, similarly to *Parkin*, confer a loss of function. Heterozygous *PINK1* and *Parkin* mutations may also be considered as a risk factor for developing PD (Khan et al., 2005; Criscuolo et al., 2006; Hedrich et al., 2006; Hiller et al., 2007).

PINK1 is a Ser/Thr kinase that is localized to the mitochondria (Hatano et al., 2004; Valente et al., 2004a,b; Zhou et al., 2008). In a normal, healthy mitochondrion PINK1 binds to the outer mitochondrial membrane (OMM), and is then imported into the inner mitochondrial membrane (IMM) *via* the translocase of the outer membrane (TOM) complex and translocase of the inner membrane 23 (TIM23) in a membrane potential-dependent manner. Full length PINK1 is then rapidly cleaved by mitochondrial processing peptidase (MPP) and presenilin-associated rhomboid-like protease (PARL). Cleaved PINK1 is released into the cytosol for other non-mitochondrial functions (Dagda et al., 2014) and can be degraded by the proteasome (Meissner et al., 2011; Yamano and Youle, 2013). Hence, PINK1 is maintained at a low level in healthy mitochondria. However, upon mitochondrial damage and the loss of membrane potential, PINK1 import is blocked causing it to accumulate at the OMM (Jin et al., 2010; Lazarou et al., 2012; Okatsu et al., 2013). At the OMM, the kinase domain of PINK1 faces toward the cytosol, enabling it to phosphorylate both mitochondrial and cytosolic proteins (Zhou et al., 2008). Although not consistently observed, PINK1 has been reported to phosphorylate ubiquitin and ubiquitin-like (Ubl) domain of Parkin at a conserved Ser65 residue. This phosphorylation initiates a cascade that consequently activates and recruits Parkin, a ubiquitin E3 ligase, to mitochondria (Durcan and Fon, 2015; Roberts et al., 2016). Activated Parkin promotes ubiquitination of multiple OMM proteins targeting them for degradation via the proteasomal pathway (Gegg et al., 2010; Chan et al., 2011; Yoshii et al., 2011; Kazlauskaite et al., 2014) as well as the recruitment of autophagy adaptor proteins (Roberts et al., 2016). The PINK1/Parkin axis is therefore an important pathway for mitochondrial quality control and the selective removal of the damaged organelle by mitophagy (Geisler et al., 2010; Narendra et al., 2010, 2012; Vives-Bauza et al., 2010).

Mitochondrial abnormalities have been reported in multiple *in vivo* and *in vitro* models of PINK1 and Parkin. PINK1 deficient and PD-associated mutant models display reduced mitochondrial membrane potential, ATP levels, reduced respiratory capacity *via* complex I and IV activity, increased mitochondrial calcium levels, sensitized mitochondrial permeability transition pore opening and increased ROS production (Gautier et al., 2008, 2012; Piccoli et al., 2008; Dagda et al., 2009; Gandhi et al., 2009; Gegg et al., 2009; Liu W. et al., 2009; Morais et al., 2009, 2014; Cui et al., 2010; Heeman et al., 2011). Isolated mitochondria from brains of aged *PINK1* null mice display complex I deficits (Gautier et al., 2008; Morais et al., 2014) and reduced calcium buffering capacity (Akundi et al., 2011). Aged *Parkin* null brains show alterations in the expression of a wide range of mitochondrial proteins, with many relating to a reduced respiratory capacity and increased oxidative damage (Palacino et al., 2004; Periquet et al., 2005; Stichel et al., 2007). PINK1 and Parkin also play a regulatory role in mitochondrial dynamics by mechanisms involving the turnover of fusion and fission associated proteins such as Mfn1/2 and Drp1 (Clark et al., 2006; Deng et al., 2008; Poole et al., 2008; Yang et al., 2008; Lutz et al., 2009; Cui et al., 2010; Gegg et al., 2010; Tanaka et al., 2010; Ziviani et al., 2010; Exner et al., 2012; Buhlman et al., 2014; Pryde et al., 2016). However, whether PINK1 and Parkin are pro-fission or pro-fusion has been a topic of debate (Exner et al., 2012; Pilsl and Winklhofer, 2012).

In addition to the above effects on mitochondrial function and dynamics, PINK1 and Parkin mediate biogenesis through an indirect interaction with PGC-1α (Shin et al., 2011; Stevens et al., 2015; Lee et al., 2017). The loss of PINK1/Parkin activity leads to a reduced clearance, and consequent build-up, of the Parkin Interacting Substrate (PARIS/ZNF746) which is a transcriptional repressor of PGC-1α. An increase in PARIS/ZNF746 is identified in the striatum and substantia nigra of patients with autosomal recessive and sporadic PD, as well as in *Parkin* knockout mice (Shin et al., 2011).

#### DJ-1

Homozygosity mapping in two consanguineous families from Italy and the Netherlands with early-onset PD identified *DJ-1* mutations (Bonifati et al., 2003). Collectively, mutations in *DJ-1* account for approximately 1–2% of early onset recessive cases of PD and encompass deletions, homozygous and heterozygous point mutations and truncations (Bonifati et al., 2003). DJ-1 is a small protein of 189 residues localized to the cytoplasm, the nucleus and associated with mitochondria (Canet-Aviles et al., 2004; Li et al., 2005; Junn et al., 2009). Among its functions, DJ-1 is involved in regulating mitochondrial activity (Hayashi et al., 2009; Junn et al., 2009) and protecting against oxidative stress (Taira et al., 2004).

DJ-1 can interact with mitochondrial complex I subunits and is translocated to the mitochondria under stress conditions (Hayashi et al., 2009). DJ-1 and its mutant forms could associate with the chaperone protein Hsp70, a link that was further increased under oxidative stress conditions. These results suggest that translocation of DJ-1 to the mitochondria may occur with the aid of mitochondrial chaperone proteins (Li et al., 2005). It is believed that the dimeric form of DJ-1 is required for antioxidative stress reactions, while monomeric mutant forms of DJ-1 are toxic for cells (Zhang et al., 2005; Maita et al., 2013). Further studies also demonstrated mitochondrial dysfunction and reduced membrane potential in *DJ-1* knockout mice and flies (Hao et al., 2010; Giaime et al., 2012). Consistent with its protective role in mitochondria, *DJ-1* knockout mice exhibit enhanced vulnerability to the mitochondrial toxicant 1-methyl-4-phenyl-1,2,3,6-tetrahydropyridine (MPTP) (Kim et al., 2005; Manning-Boğ et al., 2007).

DJ-1 is required for the proper balance of mitochondrial dynamics; primary cortical neurons and embryonic fibroblasts cultured from DJ-1 knockout mice and human dopaminergic neuronal cells with DJ-1 knockdown exhibit mitochondrial fragmentation and reduced Mfn2 levels (Irrcher et al., 2010; Krebiehl et al., 2010; Thomas et al., 2011). Blocking mitochondrial fission or promoting mitochondrial fusion rescues this abnormal mitochondrial morphology (Irrcher et al., 2010; Thomas et al., 2011). DJ-1 has also been suggested to participate in the mitophagy pathway. In fact, loss of DJ-1 leads to mitochondrial impairment and dysfunctional autophagy (Irrcher et al., 2010; Krebiehl et al., 2010; Thomas et al., 2011) and overexpression of PINK1 and Parkin is protective (Irrcher et al., 2010; Thomas et al., 2011). These results are consistent with the proposal that DJ-1 may be aiding PINK1/Parkin in the clearance of misfolded proteins and damaged mitochondria (Xiong et al., 2009; Thomas et al., 2011). Taken together, DJ-1 has a critical role in the oxidative stress response, mitochondrial function and basal autophagy.

#### PLA2G6

Mutations in the *PLA2G6* gene have been described in patients with autosomal recessive early-onset dystonia-parkinsonism with widespread accumulation of Lewy bodies and hyperphosphorylated tau (Paisan-Ruiz et al., 2009; Miki et al., 2017). Brain iron accumulation is found in most affected individuals (Paisan-Ruiz et al., 2009). The *PLA2G6* gene encodes a 85-kDa group VI calcium-independent phospholipase A_2_β (PLA2G6) and is involved in the hydrolysis of glycerophospholipids to release free fatty acids and lysophospholipids (Ma and Turk, 2001). PLA2G6 is confined to the mitochondrial compartment where it remodels membrane phospholipids, protects mitochondria from oxidative damage during apoptotic induction and takes part in calcium signaling (Gadd et al., 2006; Seleznev et al., 2006; Strokin et al., 2012). Although the pathological mechanism of PLA2G6 remains elusive, it has been suggested to affect lipid homeostasis and metabolism as well as impact mitochondrial membranes (Beck et al., 2011). Further, *PLA2G6* knockout mice displayed degenerated mitochondrial membranes and elevated levels of synuclein (Sumi-Akamaru et al., 2016). PLA2G6 has been reported to be immunopositive in the Lewy bodies of idiopathic PD, suggesting its pathogenic role extends beyond familial PD (Miki et al., 2017).

#### ATP13A2

*ATP13A2* encodes ATPase type 13A, which is usually localized to lysosomes. Mutations in *ATP13A2* were initially identified in Kufor–Rakeb syndrome (Ramirez et al., 2006), which is a recessively inherited disease with multiple brain regions are affected. Fibroblasts derived from these patients display reduced ATP synthesis, increased mtDNA mutations, impaired oxygen consumption and mitochondrial fragmentation (Grunewald et al., 2012). Mutations in ATP13A2 are also found in early onset parkinsonism (Di Fonzo et al., 2007), suggesting that ATP13A2 may also be relevant to PD. ATP13A2 is a multifunctional protein that has been linked to endosome-lysosome dynamics (Dehay et al., 2012; Usenovic et al., 2012), mitochondrial function (Grunewald et al., 2012; Gusdon et al., 2012) and toxicity induced by divalent cation metals (Mn^2+^ and Zn^2+^) (Gitler et al., 2009; Kong et al., 2014). Consistent with these roles of ATP13A2, loss of function in this protein has been reported to reduce the ability of various vesicular structures such as lysosomes to regulate divalent metal cations such as Mn^2+^ and therefore sensitize cells to metal toxicity, accumulation of α-synuclein and mitochondrial dysfunction (Gitler et al., 2009; Kong et al., 2014). These effects are most likely secondary to autophagy deficits, causing an accumulation of misfolded proteins and damaged mitochondria. Collectively, ATP13A2 may exert an indirect, protective role on mitochondria and the cell as a whole. Indeed, overexpression of ATP13A2 is protective against α-synuclein-induced toxicity (Gitler et al., 2009; Kong et al., 2014).

#### FBXO7

Mutations within the *FBXO7* (F-box only protein 7) gene have been shown to promote either a typical PD phenotype (Lohmann et al., 2015) or an early onset form of parkinsonian-pyramidal syndrome (Shojaee et al., 2008; Di Fonzo et al., 2009). FBX07 has been linked to mitochondrial function by acting on the same pathway as PINK1 and Parkin. It directly interacts with both PINK1 and Parkin and therefore plays a role in mitochondrial maintenance and mitophagy (Burchell et al., 2013; Zhou et al., 2015). These studies demonstrate that FBXO7 assists in the translocation of Parkin to the mitochondria in response to cell stress and PD-associated mutations leading to a mislocalization of FBXO7 to the cytosol where it can form toxic aggregates that are deleterious to the cell (Zhou et al., 2015).

## Environmental Exposures As Potential Links to PD

Prior to the discovery of the first genetic mutation linked to PD (Polymeropoulos et al., 1997) environmental factors were the major focus of research aiming to understand PD pathogenesis and etiology. The rationale for this dominant environmental theory was largely based on observations such as post-encephalitic infection, manganese (Mn) and MPTP exposure causing parkinsonism (Rail et al., 1981; Langston et al., 1983; Calne et al., 1994; Kwakye et al., 2015). Multiple epidemiological studies and meta-analyses have implicated a number of environmental factors, including pesticides and heavy metals, with increasing PD risk (Tanner et al., 2011; Wang et al., 2011; Kamel, 2013). These studies support the proposition that chronic exposure to environmental toxicants plays a major role in sporadic PD pathogenesis. Pesticides and heavy metals are the most widely researched environmental toxicants implicated in PD, and the ability of some of them to persist in the environment long after their application increases the likelihood of human exposure to these contaminants. Following are some environmental exposures that have been demonstrated to be toxic to the nigrostriatal pathway.

### Pesticides

#### Rotenone

A potent non-competitive mitochondrial complex I inhibitor derived from plants of the *Leguminosa* family (Soloway, 1976), rotenone is a pesticide/piscicide associated with increased risk of developing PD (Tanner et al., 2011; Kamel, 2013). Because the half-life of rotenone is relatively short (∼3 days) (Hisata, 2002), consumption of contaminated food products is an unlikely exposure route. The role of rotenone in PD is therefore most likely through low chronic exposure rather than acute toxicity. Epidemiological studies have demonstrated increased PD risk of ∼1.5–3-fold in individuals who utilized rotenone agriculturally or who lived in close proximity to its use (Tanner et al., 2011). As a highly lipophilic chemical, rotenone passes freely through the blood-brain-barrier (BBB) and cellular membranes and once inside cells it can directly alter mitochondrial function. Rotenone impairs oxidative phosphorylation by inhibiting NADH-ubiquinone reductive activity via binding to the PSST subunit of complex I of the electron transport chain (Schuler and Casida, 2001). This mechanism of rotenone is relevant to the observations that mitochondrial complex I activity is reduced in PD patients (Parker et al., 1989; Schapira et al., 1989). Rotenone has also been shown to enhance mitochondrial fission. In primary cortical neurons, rotenone induces rapid mitochondrial fragmentation within 2 h of treatment, prior to any cellular changes indicative of cytotoxicity (Barsoum et al., 2006). Altering mitochondrial dynamics, by promoting mitochondrial fusion with Mfn2 and blocking Drp1 with Drp1-K38A averts this neurotoxicity (Barsoum et al., 2006). Although the neuropathology is variable, rotenone treatment has been shown to induce motor impairment, neuroinflammation, nigrostriatal degeneration, and accumulation and phosphorylation of α-synuclein (Betarbet et al., 2000; Cannon et al., 2009) in rodent models of PD.

In addition to impairing mitochondrial function, rotenone has been demonstrated to alter mitochondrial transport and protein interactions within neurons. For example, rotenone induces microtubule depolymerisation, thereby interfering with the intracellular transport of vesicles and organelles, including mitochondria (Choi et al., 2011). A recent study revealed increased α-synuclein association with mitochondria, specifically outer membrane protein TOM20, in nigrostriatal dopaminergic neurons of rotenone-treated rats relative to controls (Di Maio et al., 2016). This observation suggests that rotenone exposure may induce α-synuclein-mediated changes in mitochondrial function and morphology to further promote PD pathology.

#### Paraquat

The herbicidal mechanism of paraquat (PQ) involves interference with photosynthesis and oxygen free radical production, resulting in damage of plant membranes (Tanner et al., 2011; Breckenridge et al., 2013). Initially based on its similar structure to MPP^+^, this widely used herbicide was hypothesized to be a potential environmental parkinsonian toxicant (Snyder and D’Amato, 1985). Epidemiological studies demonstrate that PQ exposure, both alone and in conjunction with other pesticides, increases the risk of developing PD (Costello et al., 2009; Ritz et al., 2009; Tanner et al., 2011). To further understand the mechanism by which this molecule induces dopaminergic neurotoxicity, PQ has been used experimentally to model PD (Tieu, 2011). PQ is unable to diffuse across the BBB and relies on transport by the L-neutral amino acid transporter to enter the brain (Shimizu et al., 2001; McCormack and Di Monte, 2003). Once inside the brain, PQ can enter cells through the dopamine transporter (DAT) and organic cation transporter-3 (Rappold et al., 2011). In the cytosol, PQ induces toxicity primarily through redox cycling with cellular diaphorases such as NADPH oxidase and nitric oxide synthase (Day et al., 1999), leading to the generation of superoxide. Despite its similar structure to MPP^+^, PQ is not a complex I inhibitor (Richardson et al., 2005), although this is the site where superoxide is generated from PQ redox-cycling in the mitochondria (Cochemé and Murphy, 2008). Other work also implicates complex III in the formation of paraquat-induced ROS (Castello et al., 2007). Consistent with the role of generating mitochondrial ROS, a recent study reported mitochondrial DNA damage in PD patients with prior history of exposure to PQ (Sanders et al., 2017). The mechanism by which PQ enters mitochondria, however, is currently unknown.

PQ has also been reported to increase mitochondrial fission by reducing the levels of key mitochondrial fusion factors, Mfn1/Mfn2 (Tanaka et al., 2010). In rodent models PQ has been shown to cause neuroinflammation, as well as α-synuclein upregulation and aggregation (Manning-Bog et al., 2002; Wu et al., 2005; Fernagut et al., 2007; Purisai et al., 2007). The use of PQ to model PD has been contested by some, partly because studies utilizing wild-type mice demonstrated variable results, with loss of dopaminergic nigral cell bodies but a lack of consistency in the loss of the corresponding striatal terminals (McCormack et al., 2002; Thiruchelvam et al., 2003; Tieu, 2011); however, this lack of striatal damage has been suggested to be the result of compensatory sprouting from surviving dopaminergic neurons and uptake of PQ into non-dopaminergic striatal cells. In contrast to wild-type mice, mutant mice deficient in OCT3 (*Slc22a3*), a cation transporter which can uptake PQ into cells, display enhanced striatal damage upon exposure to PQ. This increased sensitivity likely results from reduced buffering capacity by non-dopaminergic cells, leading to increased availability of PQ to damage dopaminergic terminals (Rappold et al., 2011). Despite the controversy which still surrounds the use of PQ to model PD, current evidence from epidemiological studies and mechanistic investigations appear to sustain the conclusion that PQ may increase the risk of developing PD.

#### Organochlorines

Dieldrin is one of the organochlorine pesticides that was first commercially available in 1950, with wide use in the United States until the late 1980’s. Despite decades of research into potential risk of dieldrin, it is still extensively used in many developing countries. Dieldrin has a long half-life (∼25 years) which contributes to a persistent high risk of exposure, even decades after its use has ceased. Human exposure risk may be mediated through consumption of dairy products, meat, and fish (Kanthasamy et al., 2005), although its high lipophilicity also confers risk of dermal exposure, as it can be rapidly absorbed through the skin. In addition to its environmental persistence, the ability of dieldrin to bioaccumulate, with a half-life of ∼300 days in humans, contributes to the risk it confers, as it can readily pass the BBB and amass in the brain. Indeed, dieldrin has been detected in 30% of post-mortem brain tissue of PD patients, with none in age-matched control brains (Fleming et al., 1994). Another study reported dieldrin in both control and patient brain tissue, with significantly higher levels in the caudate nucleus of PD patients (Corrigan et al., 1996). These results support a potential role of this pesticide in promoting nigral cell death and PD progression (Kanthasamy et al., 2005).

Dieldrin has been shown to exert multiple effects on mitochondria, including increased ROS production (Chun et al., 2001) and stimulation of mitochondria-mediated apoptosis (Kitazawa et al., 2003; Kanthasamy et al., 2008). Dopaminergic cells have increased sensitivity to dieldrin when compared to other cell types as demonstrated *in vitro* (Sanchez-Ramos et al., 1998; Kitazawa et al., 2001) and *in vivo* (Sharma et al., 1976; Wagner and Greene, 1978; Heinz et al., 1980). Dieldrin (and other pesticides) have also been shown to block the function of mitochondrial aldehyde dehydrogenase (ALDH class 2) (Fitzmaurice et al., 2013, 2014). ALDH2 metabolizes toxic aldehydes including 3,4-dihydroxyphenylacetaldehyde (DOPAL), which is a reactive neurotoxic metabolite of dopamine. Exposure of individuals with genetic variation in ALDH2 to these pesticides was associated with a two–sixfold increase in their risk of developing PD (Fitzmaurice et al., 2014). ALDH2 inhibition therefore has been suggested as a pathogenic mechanism in PD. Furthermore, based on a recent work that investigated the proteomic effects of dieldrin, 18 proteins involved in the respiratory chain were affected by dieldrin treatment (Cowie et al., 2017), further supporting the influence of this pesticide on mitochondrial function.

Dieldrin has also been demonstrated to directly stimulate α-synuclein fibril formation *in vitro*, by inducing a conformational change which may serve as a precursor or seed for the aggregation of further protein (Uversky et al., 2001b). Prolonged dieldrin exposure has been reported to inhibit the ubiquitin-proteasome protein degradation system (Sun et al., 2004), further promoting protein aggregation. In summary, although the mechanistic understanding remains incomplete, dieldrin has been shown to induce dopaminergic neurotoxicity through mitochondrial dysfunction, decreased protein degradation and apoptosis initiation.

#### Pyrethroids

Pyrethroids are synthetic derivatives of the naturally occurring pyrethrum from chrysanthemum flowers. These pesticides are widely used both agriculturally and in the household (Elwan et al., 2006), however, despite their classification as safe substances (Casida et al., 1983), pyrethroid intoxication in humans has been observed (Chen et al., 1991). These chemicals, which include permethrin and deltamethrin, readily cross the BBB and may accumulate at considerable concentrations within the brain (Anadón et al., 1996). Permethrin and deltamethrine have been shown to increase DAT-mediated dopamine uptake by up to 30% in the mouse striatum and induce apoptosis in dopaminergic neuroblastoma cells (Elwan et al., 2006). Because the DAT mediates neurotoxicity of some neurotoxicants (Gainetdinov et al., 1997; Donovan et al., 1999; Rappold et al., 2011), pyrethroids may enhance the vulnerability of dopaminergic neurons to toxic compounds. Furthermore, permethrin has been shown to potently inhibit mitochondrial complex I function in rat liver (Gassner et al., 1997), however, a subsequent study demonstrated that apoptosis induced by pyrethroids (Deltamethrin) is mitochondrial independent (Hossain and Weiner, 1993). Rather, deltamethrin induces calcium overload through Na^+^ channels, leading to endoplasmic reticulum stress.

A recent study investigated the potential use of permethrin to model PD in a progressive model of early life exposure to the pesticide (Nasuti et al., 2017). Treatment of Wistar rat pups with permethrin from post-natal days (PND) 6–21 induced cognitive impairments, reduced striatal dopamine levels and reduced dopaminergic neurons in the substantia nigra; all common neuropathological changes detected in PD patients. These changes were first obvious at PND 60, although no motor changes were recorded until testing at PND 150. This study adds a new potential pesticide model of PD to the repertoire currently utilized for research, and highlights an important consideration as early life exposure to some toxicants may pre-dispose individuals to pathogenic changes later in life, or initiate slow progressive changes which culminate in development of the clinical phenotype. Despite the demonstration of the neurotoxicity of pyrethroids in experimental models, there appears to be insufficient human data to link pyrethroids exposure to PD (Costa, 2015).

### Heavy Metals

Copper, iron, zinc, and Mn represent some of the heavy metals involved in the development of neurodegeneration. Collectively, these metals are required for many physiological activities, however, excessive levels may detrimentally affect cell viability through mechanisms such as mitochondrial dysfunction and oxidative stress (Martinez-Finley et al., 2013; Park et al., 2015). They can also promote DNA damage and interact with proteins that are directly involved in neurotoxicity, such as α-synuclein and β-amyloid, by altering their structures (Cervantes-Cervantes et al., 2005; Ha et al., 2007; Salvador et al., 2010; Chen et al., 2016). Taken together, these effects will eventually disrupt a variety of cellular processes and lead to increased risk of neurological damage.

Of the heavy metals implicated in PD, Mn has received the most attention. Mn is an essential metal characterized by its vital role in brain development and homeostasis (Aschner and Aschner, 2005). It has several chemical forms with 11 oxidation states and its metabolism is tightly linked to that of other heavy metals, in particular that of iron due to their analogous redox behavior (Claus Henn et al., 2011; Kim et al., 2013; Kwakye et al., 2015). Mn is essential for many enzymatic reactions; it can act as a co-factor for glutamine synthetase in astrocytes and it participates in the mitochondrial antioxidant defence system as part of the superoxide dismutase (MnSoD) (Borgstahl et al., 1992). Depending on its oxidative state, Mn can be imported to the central nervous system (CNS) through separate membrane importers such as the divalent metal transporter 1 (DMT1) (highly expressed in the basal ganglia), the DAT, calcium channels, choline and citrate transporters, transferrin, and the ZIP (Zrt- and Irt-related protein) family metal transporters (Huang et al., 2004; Gunter et al., 2013; Tuschl et al., 2013). Metal efflux also plays a critical role in maintaining the homeostatic levels of Mn; although the number of proteins involved in exporting Mn are limited compared to importer channels, they include ferroportin (Fpn), SLC30A10 (solute carrier family 30 member 10), secretory pathway Ca^2+^-ATPase 1 (SPCA1) and ATP13A2 (Tuschl et al., 2013). Chronic exposure to Mn can trigger harmful effects in the brain clinically resulting in Manganism (Couper, 1837), which is characterized by motor impairments, behavioral changes and cognitive alterations. This movement disorder has symptoms that resemble (Aschner et al., 2009), but are distinct to, those of PD (Calne et al., 1994; Guilarte and Gonzales, 2015). However, Mn exposure is believed to contribute to increased PD risk in conjunction with other factors, such as genetic susceptibility. In addition to environmental exposure, mutations in the *SLC30A10* gene (the first human Mn transporter identified) cause an accumulation of Mn in both the basal ganglia and the liver, inducing hypermanganesemia, childhood onset dystonia and adult onset parkinsonism (Quadri et al., 2012; Tuschl et al., 2012). Furthermore, in 2016, a novel autosomal recessive Mn transporter defect (*SLC39A14*) was discovered in children affected by hypermanganesemia and progressive parkinsonism–dystonia. SLC39A14 represents a specific Mn transporter as mutations in this gene impair Mn uptake, without affecting any other metal levels (Tuschl et al., 2016).

Although parkinsonism induced by Mn and PD share some similar features, it is important to note that they are two distinctive disorders (Calne et al., 1994; Guilarte and Gonzales, 2015; Kwakye et al., 2015). In fact, Mn-induced-parkinsonism does not respond to levodopa, the most commonly used and effective drug in PD. Moreover, it is believed that Mn primarily affects gamma aminobutyric acid (GABA) producing neurons in the globus pallidus while the numbers of dopaminergic neurons in the substantia nigra remain relatively unaffected; stereological cell quantification needs to be performed to confirm these results (Guilarte et al., 2006; Guilarte and Gonzales, 2015). Moreover, Guilarte et al. (2008) used Positron Emission Tomography (PET) to show that Mn-exposed animals exhibited reduced dopamine release in the dorsal striatum, demonstrating an impaired nigrostriatal dopaminergic system in Mn-exposed monkeys. Although Mn-induced parkinsonism cannot be considered as idiopathic PD, Mn can still affect PD pathogenesis by interacting with an individual’s genetic makeup as discussed below, and epidemiological study has established an accumulation of Mn levels in PD patients (Fukushima et al., 2010). In addition, chronic exposure to Mn results in significantly increased Mn levels in dopaminergic neurons of rodent brains compared to control animals (Robison et al., 2015). Therefore, it is possible that Mn can enhance the susceptibility of the individual to developing PD or accelerate the disease onset.

The precise pathological mechanism of Mn toxicity is not known, however, evidence suggests a direct interaction with mitochondria and subsequent induction of mitochondrial damage. Mn can enter mitochondria as Mn^2+^ through the Ca^2+^ uniporter channel and slow export occurs via the Na^+^-independent Ca^2+^ channel (Gavin et al., 1999). High Mn^2+^ concentrations can accumulate in the mitochondrial matrix and promote Na^+^-dependent and Na^+^-independent Ca^2+^ efflux channel through direct competitive inhibition. This subsequently causes an increase of mitochondrial Ca^2+^ levels, which interfere with oxidative respiration and induce oxidative stress (Gavin et al., 1990). It has been suggested that ROS induction can originate from superoxide which can then be converted in the mitochondria to hydrogen peroxide by the Mn and Cu/Zn superoxide dismutase. Hydrogen peroxide can be reduced to form hydroxyl radicals in the presence of Mn or other transition metals via the Fenton reaction (Goldstein et al., 1993; Martinez-Finley et al., 2013). The ROS generated by excessive Mn levels promote opening of the mitochondrial permeability transition pore, causing a loss of membrane potential, impaired ATP synthesis and mitochondrial swelling, contributing to cellular apoptosis (Gavin et al., 1990; Gavin et al., 1992). Gunter et al. (2010) measured ATP production to study how Mn^2+^ promotes inhibition of the complex oxidative phosphorylation system in multiple organs (liver, heart, and brain mitochondria). Their results showed that Mn^2+^ inhibits ATP production in different manners when comparing the different tissues. It appears that Mn^2+^ inhibits two independent sites in brain mitochondria: the primary site is electron transport chain complex II, while the second is either the glutamate/aspartate exchanger or the aspartate aminotransferase (Gunter et al., 2010).

Although previous studies have focused their attention on the effects of Mn on mitochondria and its capacity to interfere with oxidative phosphorylation, there is little evidence of the involvement of mitochondrial dynamics following Mn neurotoxicity. A recent study, however, showed that Mn induced changes in mitochondrial-shaping proteins (Opa-1, Mfn-2, and Drp-1) by shifting the balance toward mitochondrial fission (Alaimo et al., 2014). Indeed, an increased amount of Drp-1 translocation to the mitochondria was observed, indicative of increased mitochondrial fission. Mn-induced apoptosis was prevented when Drp-1 was inhibited (Alaimo et al., 2014). Furthermore, Mn toxicity could promote inflammatory events by directly modulating mitochondrial dynamics in astrocytes, and addition of mito-apocynin (a mitochondrial targeted antioxidant) significantly reduced Mn-induced inflammation (Sarkar et al., 2017). These studies demonstrate that Mn impairs mitochondrial function in both glia and neurons and that restoring mitochondrial health in either cell type is neuroprotective.

### Dietary Exposures

Diets have been documented to have an impact on the risk of developing PD. Strong epidemiological evidence indicates, for example, that cigarette smoking and coffee drinking are inversely correlated to PD development (Hernán et al., 2002). On the other hand, certain dietary exposures have also been linked to parkinsonism. Annonacin is one such example.

Annonacin is a chemical derived from plants of the *Annonacae* family, such as the soursop fruit, which has been implicated in the development of atypical parkinsonism in Guadeloupe. In the 1990’s it was observed that a high proportion of Guadeloupe’s population exhibited levodopa-resistant parkinsonism, with presentation of bradykinesia, rigidity, postural instability, and in some cases dementia (Caparros-Lefebvre and Elbaz, 1999; Caparros-Lefebvre et al., 2002). Annonacin belongs to the acetogenins, a class of large, structurally homogenous polyketides which act as potent mitochondrial poisons via inhibition of mitochondrial complex I (Tormo et al., 1999; Motoyama et al., 2002). Studies of annonacin have demonstrated its potency for complex I inhibition to be up to 50 times greater than that of MPP^+^, and at least equal to that of rotenone, consistent with its ability to cause drastic neuronal loss and the development of parkinsonian symptoms in a similar manner to these two PD-linked complex I inhibitors (Lannuzel et al., 2006). Acetogenins are highly toxic and have been shown to cause loss of dopaminergic neurons at nanomolar concentrations *in vitro* (Lannuzel et al., 2006). Due to its lipophilic nature, annonacin does not require specific transporters for entry into the brain or cells and it inhibits complex I function in both neuronal and glial populations (Degli Esposti, 1998; Lannuzel et al., 2006). Although the effects of annonacins are not specific to dopaminergic neurons, neurodegeneration of the basal ganglia is the most pronounced, with reduced alteration of the hippocampus, cerebellum and cerebral cortex (Lannuzel et al., 2006). Studies have confirmed that annonacin-treated rats exhibit neurodegeneration characteristic of PD, with stereological cell counts detailing significant loss of dopaminergic neurons in the substantia nigra, reduced dopaminergic striatal terminals and significantly increased astrocyte and microglial reactivity (Champy et al., 2004; Lannuzel et al., 2006).

Annonacin treatment of primary embryonic striatal neurons induces retrograde mitochondrial transport, with some mitochondria displaying tau protein association with their outer membranes, suggesting that annonacin may also confer risk for other neurodegenerative disorders, including Alzheimer’s disease (Escobar-Khondiker et al., 2007). The authors surmised that ATP-depletion induces this mitochondrial withdrawal from cellular extremities, which may in turn mediate loss of these axonal terminals due to insufficient fulfillment of their energy demands (Escobar-Khondiker et al., 2007). In humans with chronic exposure from fruit consumption, synergistic effects of annonacin with other acetogenins and alkaloids, which are also present in some annonacin-containing plants, may contribute to the putative toxicity of these fruits and the development of neurodegenerative diseases (Lannuzel et al., 2003; Potts et al., 2012).

### Gene–Environment Interactions in Parkinson’s Disease

As comprehension of the role of genetics and environmental exposures in the development of PD increases, it has also become clear that independently these factors cannot account for all cases of sporadic PD. For example, it has been a topic of great interest and debate why a single heterozygous mutation in the recessive *PINK1* and *Parkin* genes would lead to PD in some patients (Klein et al., 2007). It is possible that these heterozygous mutations increase susceptibility to environmental toxicants. Equally interesting is the observation that mutations in LRRK2 have also been detected in sporadic PD (Gilks et al., 2005). Additional familial PD genes have also been found among the risk loci identified for sporadic PD (Nalls et al., 2014). The view of gene–environment interactions that culminate in PD has increasingly been appreciated. However, the specifics of such interactions have not been well documented. Although some recent epidemiological studies have identified a number of specific interactions that might be relevant to PD (Cannon and Greenamyre, 2013), most results are derived from genetically modified experimental models to evaluate the sensitivity of neurons to toxicants as described below.

Rotenone has been shown to interact with or to increase PD risk due to association with multiple genes, including *Parkin* and *VPS-35*. When primary neuronal cultures from wild type or *Parkin*-knockout mice were treated with different rotenone concentrations; the *Parkin*-knockout cultures were much more sensitive to rotenone toxicity (Casarejos et al., 2006). Because Parkin also functions in the mitophagy pathway, the cumulative effects of *Parkin* defective cells with rotenone may relate to the reduced clearance of defective mitochondria. Rotenone exposure may therefore increase the likelihood of disease onset in individuals with Parkin mutations, or it may decrease the age of disease onset by potentiating the pathogenic process. Increased vulnerability of primary neuronal cultures to rotenone and *VPS35* mutations have also been reported (Tsika et al., 2014), suggesting that increased PD susceptibility due to combinatory effects between rotenone and genetic modifications is not limited to just a single gene.

Paraquat (PQ) also has a number of genetic modifications linked to increased risk and PD susceptibility, including *CHCHD2* and *PINK1* (Gegg et al., 2009; Meng et al., 2017). Viability of *PINK1*^-/-^ cells is reduced following treatment with PQ compared to control cells (Gegg et al., 2009). This enhanced vulnerability is likely due to the combination of reduced ATP production and increased intracellular oxidative stress. Increased mitochondrial dysfunction and susceptibility to apoptosis or oxidative stress over time has also been reported in brain cells with loss of PINK1 activity (Gautier et al., 2008; Wood-Kaczmar et al., 2008). *CHCHD2* is also shown to increase the sensitivity of *Drosophila* to PQ-induced oxidative stress (Meng et al., 2017), suggesting that genetic mutations which reduce the ability of cells or organisms to scavenge ROS likely increase their susceptibility to PQ neurotoxicity.

Attention has also been given to the interactions between Mn and PD-linked gene products including α-synuclein, Parkin, PINK1, DJ-1, LRRK2, ATP13A2, and VPS35, as recently discussed in these reviews (Covy and Giasson, 2011; Roth, 2014, Peres et al., 2016). Regarding its interaction with α-synuclein, Mn can bind to this protein via three residues in the C-terminal domain: Asp-121, Asn-122, and Glu-123 (Uversky et al., 2001a), although biophysical studies showed that Mn exhibits a low affinity for α-synuclein (1 mM range) compared to other metals (Binolfi et al., 2006). X-ray fluorescence imaging also indicates an interaction between α-synuclein and Mn in rat primary midbrain neurons (Dućič et al., 2015). This study reported that intracellular content of Mn was higher in cells with α-synuclein overexpression, suggesting that α-synuclein acts as an intracellular storage of Mn (Dućič et al., 2015). Perhaps partly because of this property of α-synuclein, its effects on cell viability has been reported to be both neurotoxic and neuroprotective when combined with Mn. As extensively discussed (Peres et al., 2016), although Mn has been reported by several laboratories to enhance α-synuclein aggregation and neurotoxicity when combined with α-synuclein, some studies also showed that wild type α-synuclein is protective against Mn neurotoxicity in *Caenorhabditis elegans* (Bornhorst et al., 2014) and rat dopaminergic neuronal cells (Harischandra et al., 2015). For the later study, protection was only observed during the early stages of Mn exposure, suggesting that accumulation of sequestered Mn would eventually induce protein aggregation and neurotoxicity.

Most of the current evidence on ATP13A2 function comes from studies focusing on the interaction between this protein and α-synuclein. Given its role in lysosomal degradation it is not surprising that mutations in this gene can lead to diminished lysosomal-mediated clearance of autophagosomes and accumulation of aggregated α-synuclein (Gitler et al., 2009; Usenovic and Krainc, 2012). On the other hand, ATP13A2 has been shown to protect cells against Mn toxicity (Gitler et al., 2009), however, this protection is lost when mutations in this gene occur. A recent study showed that ATP13A2 mutations could increase the detrimental effects of Mn on motor coordination in humans exposed to the heavy metal (Rentschler et al., 2012).

Mn-exposed animals displayed mitochondrial impairment and loss of tyrosine hydroxylase, a phenotypic marker for dopaminergic neurons (Sriram et al., 2010). The expression of Parkin and DJ-1 were also affected in these cells. Relevant to Mn neurotoxicity, Parkin has been reported to regulate Mn transport (Roth et al., 2010). The DMT1 transporter represents the primary route for Mn uptake in the brain and four isoforms have been identified. Parkin is involved in the ubiquitination and subsequent proteosomal degradation of one of these isoforms (Roth et al., 2010). Therefore, a loss of Parkin function could facilitate Mn transport, promoting Mn accumulation in the basal ganglia and accelerating its toxicity. *DJ-1* and Mn have further been implicated in mitochondrial dysfunction. Lee et al. (2012) showed that Mn could reduce both protein and RNA levels of DJ-1 inducing an effect that resembled the neurotoxic activity of mutant forms of DJ-1. Furthermore, the ability of both factors to individually promote oxidative stress, increase opening of the mitochondrial permeability transition pore, decrease membrane potential and impair ATP represent possible exacerbating effects of Mn and DJ-1 on mitochondrial activity. In summary, Mn has been demonstrated to interact with many PD-linked gene products and dysregulation of Mn homeostasis or exposure to Mn may contribute to PD pathogenesis.

## Conclusion and Future Directions

First described in 1817, PD is now recognized as a major debilitating neurological disorder. To develop effective disease modifying therapies or a cure, it is critical to understand what causes PD and its associated pathogenic mechanism(s). Until the first PD linked mutation was discovered in 1997, the search for the cause of PD was primarily focused on environmental factors. This approach seems logical given the absence of genetic evidence at the time and the observations that PD/parkinsonism could be caused by post-encephalitic infection and exposure to pesticides, Mn, MPTP and diets as discussed in this review. However, after the first discovery PD-linked mutation, PD research has progressed at a rapid pace with exciting directions. In the last few decades, we have learned tremendously about this disease. However, the more we know, the more we realize how complex this disorder truly is. To date, no other neurodegenerative disorders exhibit so many genetic mutations (and yet they represent only a small fraction of all cases), and with such a strong environmental component as PD. The etiology of PD is complex and multi-factorial.

**FIGURE 1:**
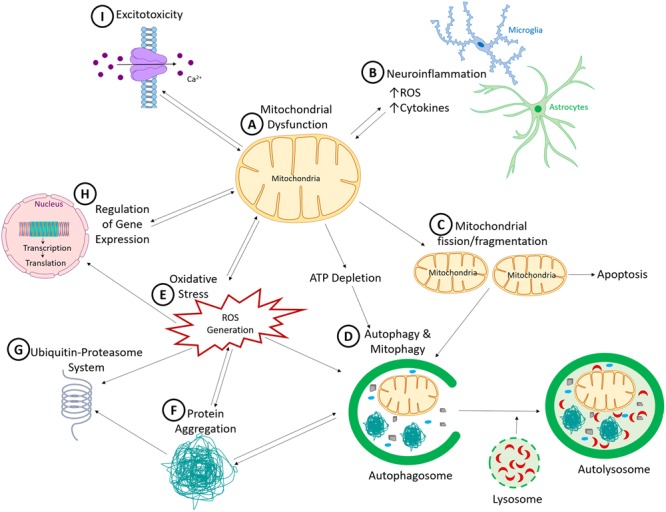
Common pathogenic mechanisms in Parkinson’s disease. **(A)** Mitochondrial dysfunction, a common pathogenic mechanism induced by many of the environmental toxicants and genetic mutations linked to PD, results in a cascade of interconnected cellular dysfunction. **(B)** Neuroinflammation, facilitated by microglia and to a lesser extent astrocytes, which release neurotoxic factors such as cytokines, interleukins and reactive oxygen species, leading to non-cell autonomous neurotoxicity. **(C)** Increased mitochondrial fission and fragmentation, which can initiate apoptotic cell death by inducing cytochrome C release. **(D)** Because autophagy and ubiquitin-proteasome system (UPS) are ATP-dependent processes, ATP reduction would reduces autophagic clearance of damaged proteins and organelles. This process is also sensitive to reactive oxygen species (ROS). **(E)** Generation of ROS, which has the capacity to promote the formation of toxic oligomers and protein aggregates **(F)**, impair ubiquitin proteasomal system function **(G)**, and induce DNA damage (both nuclear and mitochondrial DNA). **(H)** Nuclear DNA encodes numerous mitochondrial proteins. DNA damage results in altered nuclear function, genomic instabilities, and mitochondrial dysfunction. **(I)** Dysregulated cellular Ca^2+^ is influenced by mitochondrial dysfunction, because mitochondria help to regulate intracellular Ca^2+^ levels. When damaged, mitochondria release more Ca^2+^ into the cytosol, thereby increasing cellular excitotoxicity. Over-activation of the excitatory receptors also results in excitotoxicity due to Ca^2+^ influx which then produces downstream defects such as Ca^2+^-induced mitochondrial depolarization. As illustrated, all these mechanisms cross-talk and culminate in neurodegenerative processes in PD.

What have we learned so far about PD etiology and pathogenesis? We have identified several autosomal dominant and recessive genes linked to familial PD, as well as those that increase the risk of PD. The successive and rapid discoveries of these genes have led many PD researchers to shift their focus to genetic studies. However, we have now reached a stage where we realize that even with additional genes to be discovered in the future, genetics alone cannot account for all PD cases. Data from epidemiological and genome-wide association studies support the role of environmental factors and gene–environment interactions in sporadic PD. Experimental models also reveal that all three factors (genetic, environmental and gene–environment interaction) have an impact on the nigrostriatal pathway. In combination, these studies have provided significant insights into mechanisms of neuronal dysfunction and degeneration in PD. These non-mutually exclusive mechanisms include mitochondrial dysfunction, oxidative stress, neuroinflammation, toxic misfolded proteins, and insufficient protein degradation (as illustrated in **Figure 1**).

**FIGURE 2:**
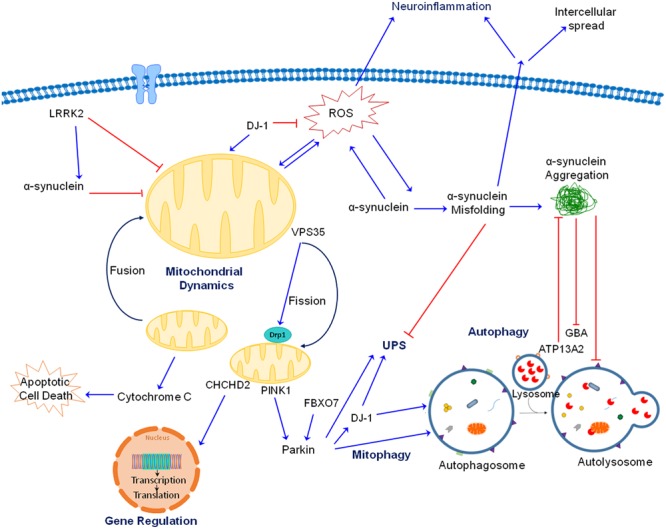
Interactions between gene products linked to PD. As discussed in the text, both genetic mutations and neurotoxicants linked to PD impair mitochondrial function, whether directly or indirectly by interacting with each other. For the autosomal recessive gene products, PINK1 is accumulated at outer mitochondrial membrane (OMM) of damaged mitochondria, where it recruits Parkin which ubiquitinates proteins and target them for degradation. Together, PINK1 and Parkin play an important role in maintaining mitochondrial quality through mitophagy. As an antioxidant protein, DJ-1 is translocated to dysfunctional mitochondria when ROS is generated. DJ-1 also aids PINK1 and Parkin in removing damaged mitochondria. Additionally, FBX07 is involved in the recruitment of Parkin to damaged mitochondria and subsequent mitophagy. ATP13A2 is localized in lysosomes and is important for protein degradation. For the autosomal dominant gene products, accumulation of misfolded and aggregated α-synuclein can be induced by a variety of neurotoxicants and genetic mutations. Toxic α-synuclein can impair mitochondrial function, generate ROS and block autophagy/lysosomal function. Mutation in LRRK2 results in gain of toxic kinase function which impairs mitochondrial and autophagic function as well as promotes α-synuclein aggregation. VPS35 mutations increase binding to Drp1 resulting in mitochondrial fragmentation. Of note, mitochondrial fragmentation is a common observation induced by genes and toxicants linked to PD. CHCHD2 is rather unique because it is both a mitochondrial protein and transcription factor. Its mutation impairs complex IV function and expression of a complex IV subunit. Mutations in GBA, a lysosomal protein, impairs protein degradation and mitochondrial function. Although not shown for simplicity, neurotoxicants such as Mn also interact with these proteins (such as α-synuclein) to induce neurotoxicity. Together, there are extensive interactions between these proteins and neurotoxicants leading to neurodegeneration.

In this review we focus our attention on the role of mitochondrial dysfunction. By examining all the major genetic mutations and environmental toxicants linked to PD, it is evident that mitochondria are a common pathogenic target (**Figure 2**). Given the extensive and critical role of mitochondria, ranging from the classic “powerhouses” of the cell, to maintaining calcium homeostasis, controlling synaptic activity, initiating apoptosis and the spread of α-synuclein, it is not surprising that impaired mitochondrial function would negatively impact neuronal function and viability. The proposal that mitochondria represent a common and convergent pathway for PD raises some questions that might be worthwhile for future research:

First, would therapeutically targeting mitochondria alone be sufficiently efficacious? Given that other pathogenic mechanisms (oxidative stress, neuroinflammation, protein aggregation, and kinase activity) also occur in PD and that the mitochondrion is not always the first neurotoxic target, perhaps a “cocktail” therapy, a strategy used for HIV and cancer treatment, is necessary. Second, regardless of whether mitochondrial impairment is induced by environmental toxicants or genetic mutations, multiple cell types in various brain regions would be affected. Yet, dopaminergic neurons are the most vulnerable cell type. Understanding what dictates this selective cell death not only will shed light on disease pathogenesis but also therapeutic strategy. It has been proposed that this process involves a combination of selective expression of L-type Ca^2+^ channel leading to elevated cytosolic Ca^2+^, oxidized cytosolic dopamine, α-synuclein and the inherent long, highly branched axons of nigral dopaminergic neurons (Obeso et al., 2017). Although attractive, this hypothesis remains to be validated. Third, in addition to mitochondrial dysfunction, another theme gaining significant momentum is the autophagic impairment pathway. Although limited, evidence exists to support that these mechanisms, mitochondria and autophagy, are not mutually exclusive and they can bi-directionally regulate each other. It is not clear, however, which one would play a more prominent role. Fourth, emerging evidence seems to support the idea of blocking mitochondrial fission as a therapeutic strategy for PD and other neurodegenerative disorders. However, because a balance of mitochondrial fission/fusion is critical to normal physiological process, how to target this pathway to achieve optimal therapeutic with minimal side effects will require more efforts. In summary, mitochondrial dysfunction is a pathogenic mechanism shared by genetic mutations and environmental toxicants linked to PD. However, substantial further research is required before a safe and effective disease-modifying therapy may be translated into the clinical setting.

## Author Contributions

MH, JP, CS, and KT wrote the manuscript. KT edited the manuscript.

## Conflict of Interest Statement

The authors declare that the research was conducted in the absence of any commercial or financial relationships that could be construed as a potential conflict of interest.
